# Stormwater runoff drives viral community composition changes in inland freshwaters

**DOI:** 10.3389/fmicb.2014.00105

**Published:** 2014-03-14

**Authors:** Kurt E. Williamson, Jamie V. Harris, Jasmin C. Green, Faraz Rahman, Randolph M. Chambers

**Affiliations:** Department of Biology, The College of William and MaryWilliamsburg, VA, USA

**Keywords:** viruses, RAPD-PCR, community ecology, particle-associated, freshwater, terrestrial runoff, sediment resuspension

## Abstract

Storm events impact freshwater microbial communities by transporting terrestrial viruses and other microbes to freshwater systems, and by potentially resuspending microbes from bottom sediments. The magnitude of these impacts on freshwater ecosystems is unknown and largely unexplored. Field studies carried out at two discrete sites in coastal Virginia (USA) were used to characterize the viral load carried by runoff and to test the hypothesis that terrestrial viruses introduced through stormwater runoff change the composition of freshwater microbial communities. Field data gathered from an agricultural watershed indicated that primary runoff can contain viral densities approximating those of receiving waters. Furthermore, viruses attached to suspended colloids made up a large fraction of the total load, particularly in early stages of the storm. At a second field site (stormwater retention pond), RAPD-PCR profiling showed that the viral community of the pond changed dramatically over the course of two intense storms while relatively little change was observed over similar time scales in the absence of disturbance. Comparisons of planktonic and particle-associated viral communities revealed two completely distinct communities, suggesting that particle-associated viruses represent a potentially large and overlooked portion of aquatic viral abundance and diversity. Our findings show that stormwater runoff can quickly change the composition of freshwater microbial communities. Based on these findings, increased storms in the coastal mid-Atlantic region predicted by most climate change models will likely have important impacts on the structure and function of local freshwater microbial communities.

## Introduction

Viruses are the most abundant organisms in aquatic systems, typically outnumbering bacteria 10- to 100-fold, and the majority of environmental viruses are believed to infect prokaryotes (Wommack and Colwell, 2000; Weinbauer, 2004). Viral infections can impact microbial community function by influencing abundance and species composition of host communities (Thingstad, 2000; Bonilla-Findji et al., 2009; Sandaa et al., 2009). The release of dissolved organic compounds from viral lysis of cells can also influence global nutrient cycles (Azam et al., 1983; Bratbak et al., 1992; Fuhrman, 1999; Bonilla-Findji et al., 2008). In pelagic marine waters, viruses are thought to infect primarily co-occurring bacterial hosts (Proctor and Fuhrman, 1990). Novel virus types may be introduced into freshwater systems via groundwater discharge (Yates et al., 1985; Abbaszadegan et al., 1993), sewage outfall (Krikelis et al., 1986), or land-based stormwater runoff (Rajal et al., 2007).

Storms and land-based stormwater runoff can significantly impact coastal and inland waters. Runoff conveys an astonishing array of materials from terrestrial sources to aquatic sinks. A partial list of stormwater runoff components includes solids such as sand, silt, clay, particulate organic matter, gravel, and trash; chemicals, including nitrogen and phosphorus compounds, soluble organic matter, pesticides, hydrocarbons; metals such as Zn, Pb, Hg, Fe; and microbes, including protozoa, bacteria, and, as mentioned above, viruses (U.S. EPA, 2007, 2008). Research regarding the impacts of microbes in stormwater runoff on receiving waters has historically focused on human pathogens (Geldreich, 1996; Haile et al., 1999; Ferguson et al., 2003; Arnone and Walling, 2007; Davies et al., 2008; Viau et al., 2011; Teng et al., 2012; Shapiro et al., 2013). Typically, fecal coliform plate counts (Knight et al., 2000; Jeng et al., 2005; Schoonover and Lockaby, 2006) or PCR detection of specific viral nucleic acid sequences (Rajal et al., 2007) are used to assess the microbiological quality of runoff-impacted waters, and identify potential threats to human health.

To the best of our knowledge, there has been only one investigation into the impacts of runoff on natural communities of viruses in freshwaters. Hewson et al. determined the potential contribution of terrestrially derived viruses to the viral communities of two freshwater lakes in New York state (Hewson et al., 2012). The research team constructed two metagenomic libraries for each lake, one representing surface water and one representing catchment soils. After sequence analysis of each library, specific sequences were selected to serve as indicators of viral origin, with certain sequences associated with terrestrial viruses, and certain sequences associated with aquatic viruses. By designing qPCR primers for these two groups of viruses, the team tracked the abundance of each virus group in the two lakes over a 5 week monitoring period. Aquatic virus sequences amplified consistently across all samples during the observation period, but the terrestrial virus sequences were only detected after rainfall-runoff events. These results provide strong evidence that stormwater runoff regularly introduces potentially novel virus types to aquatic habitats. However, the impacts of stormwater runoff on aquatic viral community dynamics and composition are still poorly understood.

The main goal of this paper was to determine how freshwater viral communities respond to the specific disturbance represented by storms and the influx of stormwater runoff. We first estimated the actual microbial abundance carried in stormwater runoff by sampling directly in an erosion channel during a storm. We then conducted field studies in a stormwater retention pond over the courses of two major storms (Hurricane Sandy and Tropical Storm Andrea) to quantify changes in the viral community, including the particle-associated fraction, due to stormwater runoff. Viral abundance was measured using epifluorescence microscopy and changes in viral community composition were determined using RAPD-PCR. The community and environmental data were then analyzed to identify specific environmental factors that best explained observed changes.

## Materials and methods

### Study sites and sample collection

#### Site 1: charles city county (CCC) plantation

**Figure 1:**
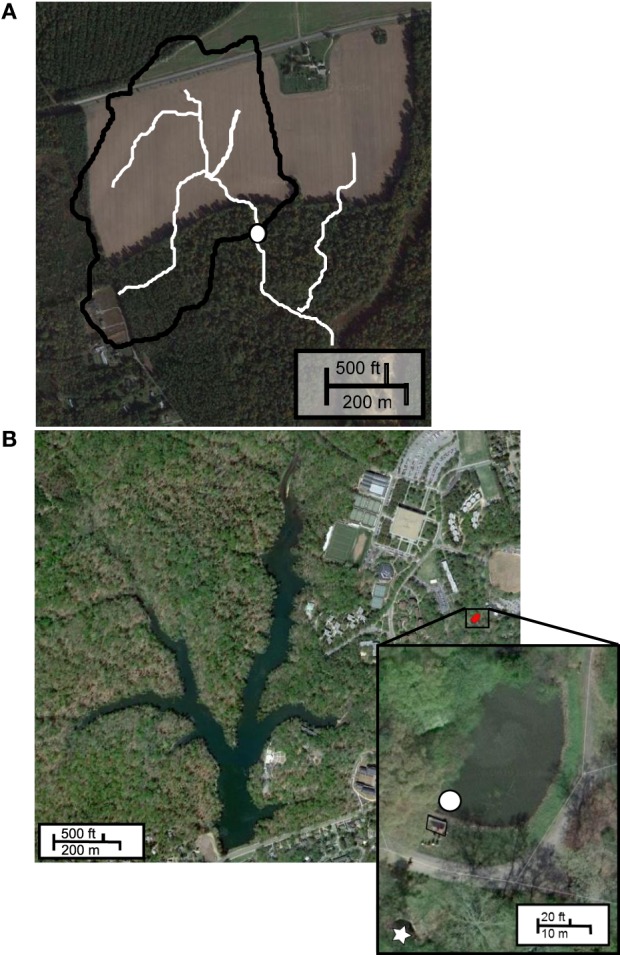
**Sample sites. (A)** CCC Plantation, white circle indicates sampling location, white lines indicate flow pathways; **(B)** Grim Dell stormwater retention pond (inset), white circle indicates sampling site, box indicates concrete weir, white star indicates outflow.

Owing to the diffuse nature of non-point source runoff, sampling can be problematic. To assess the microbial load carried by surface runoff, we chose to collect and analyze water from an ephemeral channel draining a 21-ha agricultural watershed at CCC Plantation, Charles City, VA (Figure 1A). Channel water was collected at discrete time intervals during Tropical Storm Lee (September 6–8, 2011) in polypropylene bottles using an ISCO 3700 automated sampler, and stored at −20°C following retrieval. These samples were kindly provided by James M. Kaste and Gregory S. Hancock, Department of Geology, College of William and Mary.

#### Site 2: Grim Dell

The “Grim Dell” (Figure 1B, inset) is a 2316 m^2^ wet stormwater retention pond (max. depth 2.37 m) on the William and Mary campus. The pond drains a 22.77 ha watershed and discharges to Crim Dell creek and Lake Matoaka. Surface water samples were collected near the concrete weir (37°16'16.8” N, 76°42'59.9” W) in polypropylene bottles using an ISCO 3700 automated sampler (Teledyne Isco, Lincoln, NE) during “Superstorm” Sandy (October 26–November 1, 2012) and stored at −20°C until analysis (approximately 1 month). Surface water samples were collected by hand in sterile polycarbonate bottles during Tropical Storm Andrea (June 3–14, 2013) and during a comparatively dry period (July 16–19, 2013). All samples were immediately frozen in liquid nitrogen and stored at −80°C until analysis (approximately 1 month).

### Viral and bacterial abundance

Water samples (2 ml) were dispensed into cryovials, flash-frozen in liquid nitrogen, and stored at −80 C until use (Wen et al., 2004). Viral and bacterial abundances were determined as described in Hardbower et al. (2012). Briefly, dilutions of thawed, whole water samples were passed through 0.02 μm Anodisc filters (Whatman, Maidstone, England), stained with 1× SYBR Gold (Life Technologies, Grand Island, NY), and bacterial cells and virus-like particles were enumerated using epifluorescence microscopy. Particle-associated microbial abundances were determined by centrifuging 200 ml of whole water at 8000 ×g for 20 min at 4°C to collect total suspended solids (TSS). The supernatant was decanted and the pellet was resuspended in 50 ml sterile potassium citrate buffer (Williamson et al., 2013). The slurry was sonicated in an ice bath 3 × 1 min at 60 W using a Branson 250 sonication probe outfitted with a \textonequarter” micro-tip, with 30 s of manual shaking in between each round of sonication (Danovaro et al., 2001; Williamson et al., 2007). Aliquots were then diluted 100- to 250-fold and microbial abundances were determined as described above. Subsamples (50 ml) of the sonicated slurry were centrifuged at 5000 ×g for 20 min to pellet suspended solids and the supernatant was filtered through 0.22 μm Sterivex syringe filters (Millipore, Billerica, MA) to remove bacteria. Viral concentrates (VCs) were prepared as described below.

### Water chemistry and physical properties

A YSI-63 hand-held multimeter was used to measure temperature and conductivity and a YSI-55 hand-held probe was used to measure dissolved oxygen in the field (YSI Inc., Yellow Springs, OH). Nutrient concentrations were determined through colorimetric assays using GF/F (glass fiber)—filtered water (Parsons et al., 1984). Rainfall data were obtained from the National Atmospheric and Oceanic Administration (http://www.noaa.gov/wx.html).

### Analysis of viral community composition by RAPD-PCR

VCs were prepared using ultracentrifugation as in Hardbower et al. (2012). Aliquots (50 ml) of water samples were filtered through 0.22 μm Sterivex syringe filters (Millipore, Billerica, MA) into polyallomer ultracentrifuge tubes (Beckman-Coulter, Pasadena, CA). Tubes were spun at 22,000 rpm for 2 h at 4°C in a Beckman SW41 Ti rotor to pellet virus particles. Supernatants were carefully decanted to avoid disturbing viral pellets, and pellets were resuspended in 20–50 μl TMG buffer (10 mM Tris-Cl, 10 mM MgSO_4_, 1% glycerol). Sodium azide (0.1% final conc.) was added to inhibit growth of any potential bacterial contamination. VCs were confirmed to be free of microbial contaminants by epifluorescence microscopy as previously described (Helton and Wommack, 2009; Winter and Weinbauer, 2010; Hardbower et al., 2012) VCs were stored at 4°C until use, generally within 1 month of sample collection. RAPD-PCR reactions were set up using primer CRA-22 (5′-CCGCAGCCAA-3′) and thermocycler conditions were programmed as described in Winget and Wommack (2008). Products from RAPD-PCR were separated by gel electrophoresis on 13 × 16 cm 1.8% MetaPhor agarose gels (Lonza, Aplharetta, GA) in 0.5× TBE buffer, run at 4 V cm^−1^. Gels were stained with 1× SYBR Safe (Invitrogen, Eugene, OR) in 0.5× TBE for 1 h prior to visualization of bands using a Kodak Gel Logic 100 imaging system. Banding patterns were analyzed using ImageQuant TL software (GE Life Sciences, Piscataway, NJ) and converted to a binary matrix representing the presence-absence of viral operational taxonomic units (OTU) (Hardbower et al., 2012).

### Statistical analyses

Environmental data were checked for normality using D'Agostino and Pearson omnibus normality test; viral and bacterial abundance values failed the test and were log_10_-transformed to obtain normal distributions. Binary matrix data representing the presence-absence of viral taxa (RAPD banding patterns) were converted into dissimilarity matrices (Dice method). Detrended correspondence analysis (DCA) of viral community matrices and environmental data matrices was performed using R (v.2.12.1). For all data sets, the length of the first DCA axis was <2 and, thus, a linear relationship between species and environmental variables was assumed (Jongman et al., 1995). To determine the most suitable set of parameters that explained viral abundance, bacterial abundance, and viral richness, stepwise multiple regression analyses were performed. Multicollinearity of multiple regression models were evaluated by calculating the variance inflation factor (VIF) as given by VIF_*j*_ = 1/1 − *R*^2^_*j*_, using package fmsb in *R*. VIF values (<5 in all cases) showed that the stepwise multiple regression analysis was not affected by collinearity of the parameters. The effects of the parameters on the multiple regression models having a *p*-value <0.05 were assumed to be significant. Relationships between viral community data and explanatory environmental variables were analyzed by redundancy analysis (RDA). Environmental variables best describing changes in viral community composition were identified by forward selection. Explanatory variables were added until further addition of variables failed to contribute to a significant improvement to the model's explanatory power.

Dice dissimilarity matrices representing viral community data were analyzed using non-metric multidimensional scaling (NMDS) in PAST (Hammer et al., 2001). NMDS was used to detect patterns that could explain the observed similarities or dissimilarities (distances) among samples. In NMDS plots, the closer two samples are plotted together, the more similar their viral community compositions, and the more distant two samples are from each other, the more dissimilar their viral community composition. The lower the stress value, the better the goodness of fit for the overall model.

Multivariate regression trees (MRTs) were used to determine the degree to which time and different environmental factors (explanatory variables) were predictive of the viral community composition (response variable). MRT is a robust predictive method even when high-order interactions exist among explanatory variables (De'ath, 2002), thus it is appropriate to our analysis. MRTs were constructed using the mvpart package in R with 100 cross-validations to select the best tree. The tree is constructed by repeated binary splitting of the data, where each split is defined by a simple rule, usually based on one to a few explanatory variables, and forms two nodes. Splits are chosen to maximize the homogeneity of the resulting two nodes. The terminal nodes (leaves) represent the groups of data formed by the tree. The depth of the tree following each split is proportional to the variance explained by the split, and the cross-validated relative error is an indicator of the tree's value in predicting changes in the response variable, where 0 = perfect prediction and 1 = no predictive value.

## Results

### CCC plantation: viral load carried by stormwater runoff

**Figure 2:**
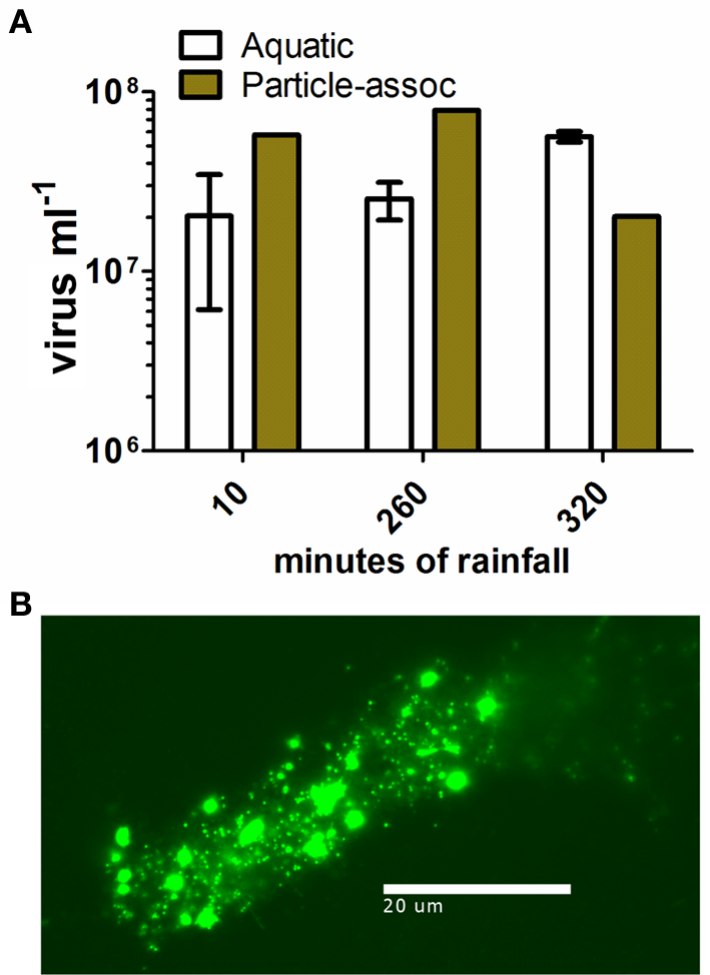
**Viral abundance and particle-associated viral abundance in stormwater runoff. (A)** Mean viral abundance in channel water sampled at CCC Plantation. Open bars, aquatic virus concentration, error bars represent SD (*n* = 2); closed bars, colloidal particle-associated virus concentration (*n* = 1); **(B)** epifluorescence micrograph showing colloid-associated microbes in stormwater runoff.

Tropical Storm Lee delivered 214.12 mm of rainfall over 67 h and a total of 12,700 m^3^ of stormwater passed through the erosion channel at the CCC Plantation site during the observation period (Caverly et al., 2013). Three water samples, collected directly within the ephemeral channel, were analyzed to determine the viral load carried by stormwater runoff. The concentration of planktonic viruses varied between 1 × 10^7^ and 5 × 10^7^ ml^−1^ (Figure 2A). Stormwater runoff also contained many suspended particles with microbes attached (Figure 2B). The particle-associated viral abundance within stormwater runoff was 2.02–7.8 × 10^7^ ml^−1^, comprising 26.4–75.6% of the total viral abundance in the runoff (Figure 2A). Given the concentrations of viruses in stormwater runoff, including both the planktonic and particle-associated fractions, and the volume of water flux during the storm, approximately 10^15^ virus particles passed through the erosion channel during this single storm event.

### Response of Grim Dell retention pond to hurricane sandy

Surface water samples were collected at regular 6-h intervals for the duration of Hurricane “Superstorm” Sandy (October 26–November 1, 2012). During the 138-h observation period, rainfall began at 34.5 h and averaged about 2 mm h-1 over the next 36 h (Table 1). Peak rainfall occurred at 72 h, with almost 28 mm of rain in the preceding 6-h period. Rainfall then tapered off, concluding by 108 h into the observation period. Water pH varied from a low of 6.17 after peak rainfall to a high of 6.84 just prior to the beginning of rainfall (Table 1). Conductivity followed a similar trend with the lowest value (73 μS cm^−1^) occurring just after peak rainfall and the highest values (>700 μS cm^−1^) observed prior to rainfall. Ammonium concentrations varied from below the limit of detection to a maximum of 10.1 μM at the 6 h time point, but showed no clear trend over time or with regard to rainfall. Phosphate concentrations were generally lower at the beginning and end of the observation period and higher during rainfall, peaking at 4.5 μM. Nitrate and nitrite concentrations were generally lower during the beginning of the observation period, ranging from 0.5 to 2.8 μM for the first 66 h, then increasing to a maximum concentration of 12.9 μM after peak rainfall. The high levels of TSS in the first three time points were due to improper positioning of the ISCO sampler intake too close to bottom sediments. Taking this into consideration, suspended solids varied from a low of 6 mg l^−1^ just prior to the beginning of rainfall, with the maximum concentration of 102 mg l^−1^ coinciding with peak rainfall, and steadily decreasing concentrations to the end of the observation period.

**Table 1:** **Environmental and community data for Grim Dell retention pond during Hurricane Sandy (October, 2012)**.

**Date (mm/dd/yyyy)**	**Time (h)**	**Conductivity (μS cm^−1^)**	**pH**	**PO_4_ (μM)**	**NH_4_ (μM)**	**NO_2_ + NO_3_ (μM)**	**TSS (mg L^−1^)**	**Partic P (μM)**	**Prior 6 h rainfall (mm)**	**Viral richness**
10/26/2012	0	737	6.68	0.18	2.82	0.5	189*	4.63	0	11
10/26/2012	6	705	6.74	0.22	10.05	1.9	87*	3.04	0	ND
10/27/2012	12	702	6.82	0.18	1.32	1.5	37*	1.74	0	11
10/27/2012	18	717	6.83	0.40	2.47	0.5	17	1.77	0	ND
10/27/2012	24	718	6.83	0.35	3.09	0.8	12	1.74	0	10
10/27/2012	30	702	6.84	0.40	1.63	3.4	10	1.56	0	ND
10/28/2012	36	699	6.84	0.44	0.79	3.4	6	1.26	0.25	10
10/28/2012	42	288	6.68	1.37	1.50	0.4	27	3.80	12.5	7
10/28/2012	48	131	6.60	1.11	BDL	0.5	20	2.07	13.19	7
10/28/2012	54	107	6.48	3.59	BDL	0.7	16	1.66	12.38	ND
10/29/2012	60	113	6.39	4.03	BDL	0.8	14	1.56	8.78	9
10/29/2012	66	101	6.39	3.37	3.53	2.8	28	1.60	12.11	10
10/29/2012	72	76	6.41	3.23	1.81	4.8	44	2.19	27.9	11
10/29/2012	78	73	6.31	4.56	0.31	6.3	102	2.76	19.54	7
10/30/2012	84	86	6.27	3.85	4.98	6.1	73	2.10	8.02	10
10/30/2012	90	109	6.23	3.37	0.04	5.6	68	1.97	2.5	10
10/30/2012	96	116	6.21	3.37	BDL	6.2	53	1.71	2	ND
10/30/2012	102	175	6.17	2.97	1.19	9.2	59	1.60	0.25	ND
10/31/2012	108	214	6.17	2.35	0.66	12.9	48	1.34	0	7
10/31/2012	114	259	6.25	1.86	BDL	5.6	34	1.34	0	ND
10/31/2012	120	259	6.26	2.39	0.04	7.3	24	1.22	0	10
10/31/2012	126	377	6.29	1.55	1.15	6.1	18	1.30	0	ND
11/1/2012	132	381	6.30	2.08	2.38	4.4	18	1.40	0	ND
11/1/2012	138	432	6.28	1.24	6.39	8.0	13	1.32	0	11

TSS, total suspended solids; asterisk indicates questionable reading due to sample probe initially lodged in sediment; Partic P, particulate phosphorus; BDL, below detection limit; ND, not determined.

**Figure 3:**
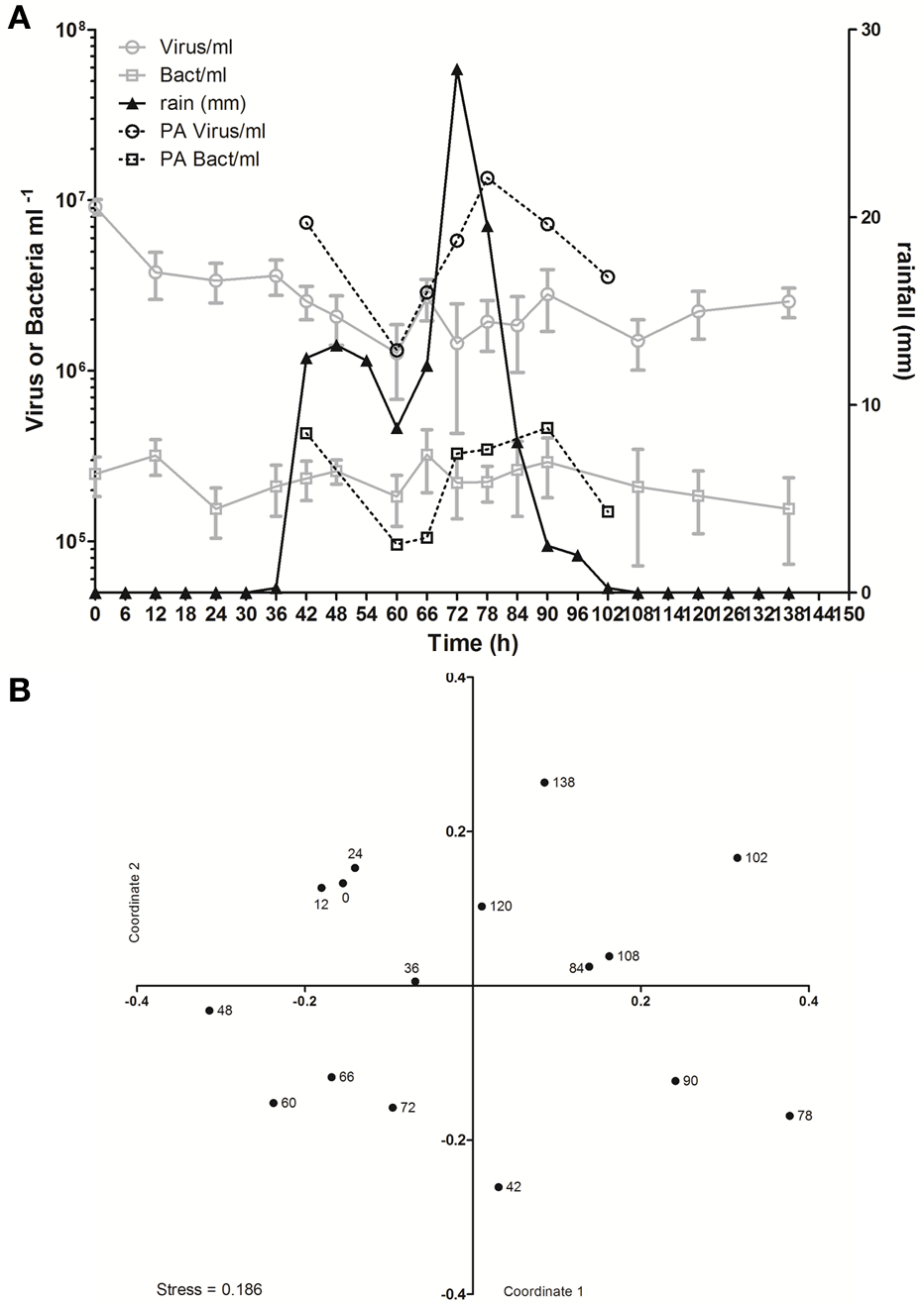
**Viral dynamics in Grim Dell retention pond during Hurricane Sandy. (A)** Mean abundances of aquatic viruses (gray circles) and bacteria (gray squares), particle-associated viruses (black circles) and bacteria (black squares, left axis), and rainfall (black triangles, right axis); error bars for aquatic viral and bacterial abundances represent SD (*n* = 2). **(B)** Non-metric multidimensional scaling (NMDS) plot of viral community composition as determined by RAPD-PCR banding patterns based on Dice dissimilarity. Data points are coded by number of hours elapsed in the observation period and correspond to samples listed in Table 1.

In spite of the large amount of precipitation, microbial abundance in the pond only varied by a factor of about 3 over the observation period, with viral abundance ranging from 1.4 to 3.6 × 10^6^ ml^−1^ and bacterial abundance ranging from 1.5 to 3.2 × 10^5^ ml^−1^ (Figure 3A). Viral abundance was consistently higher than bacterial abundance by a factor of about 10. Stepwise multiple regression analysis indicated that variation in viral abundance was best explained by the combined effects of conductivity, pH, and bacterial abundance (*R*^2^ = 0.85, *p* < 0.001), but no suitable model was found to explain changes in bacterial abundance (Table 2).

**Table 2:** **Stepwise multiple regression analysis of biological response variables in relation to environmental variables for Grim Dell retention pond during Hurricane Sandy (October, 2012)**.

**Parameters**	**Adjusted *R*^2^**	***F***	**Coefficient**	***SE***	***p***
Viral abundance	0.848	16.65			<0.001
Cond			0.009	0.0001	<0.001
BA			0.720	0.243	0.016
pH			−0.623	0.253	0.039
Bacterial abundance					
None					ns
Viral richness	0.599	4.49			0.027
Cond			0.017	0.003	0.001
pH			−17.1	4.03	0.002
NO_2_ + NO_3_			−0.522	0.157	0.01
Partic P			−1.12	0.363	0.01
Rain			0.188	0.617	0.01

Parameters defining the best performing models are shown.

Cond, conductivity; BA, bacterial abundance; Partic P, particulate phosphorus; Rain, rainfall; SE, standard error; ns, not significant.

Based on the observation from the CCC Plantation samples that runoff contains high concentrations of particle-associated viruses, a subset of the Grim Dell time series was analyzed to determine the abundances of particle-associated microbes (Figure 3A). Particle-associated viral abundances varied from 1.32 × 10^6^ to 1.35 × 10^7^ ml^−1^ and accounted for 50.9–87.4% of the total viral abundance within a given sample, while particle-associated bacterial abundances varied from 9.6 × 10^4^ to 4.6 × 10^5^ ml^−1^, comprising 24.5–64.8% of the total bacterial abundance. Not surprisingly, peaks in the concentration of TSS coincided with peaks in the abundance of particle-associated viruses and bacteria (Table 1, Figure 3A). As observed in the CCC Plantation samples, the abundances of particle-associated microbes during the storm were generally, but not always, higher than the abundances of planktonic microbes. Spearman correlations were performed on a subset of the data for which particle-associated viral and bacterial abundances were available. Strong and significant relationships were identified between particle-associated viruses and particle-associated bacteria (*r* = 0.857, *p* = 0.024), between particle-associated viruses and particulate phosphorus (*r* = 0.929, *p* = 0.007) and between particle-associated bacteria and particulate phosphorus (*r* = 0.786, *p* = 0.045).

VCs were prepared from a subset of all samples and viral community composition was compared based on RAPD-PCR banding patterns. Band richness varied from 7 to 11 distinct band types (Table 1). Stepwise multiple regression analysis indicated that viral richness was best explained by combined effects of conductivity, pH, nitrate + nitrite, and particulate phosphorus, although this model was not particularly robust (*R*^2^ = 0.60, *p* = 0.027; Table 2). NMDS analysStepwise multiple regression is of changes in RAPD banding patterns demonstrated that disturbance from Hurricane Sandy resulted in changes in viral community composition (Figure 3B). The first three time points show relatively little change in viral community composition over the 18 h prior to rainfall. At 34.5 h, rainfall begins and at 36 h the community composition begins to shift more dramatically. Large shifts are associated with the initial rainfall (36–42 h), and following more intense periods of rain (72–84 h, Figure 3B). Following the conclusion of the storm, the viral community continued to change over time up to the 138 h time point.

**Figure 4:**
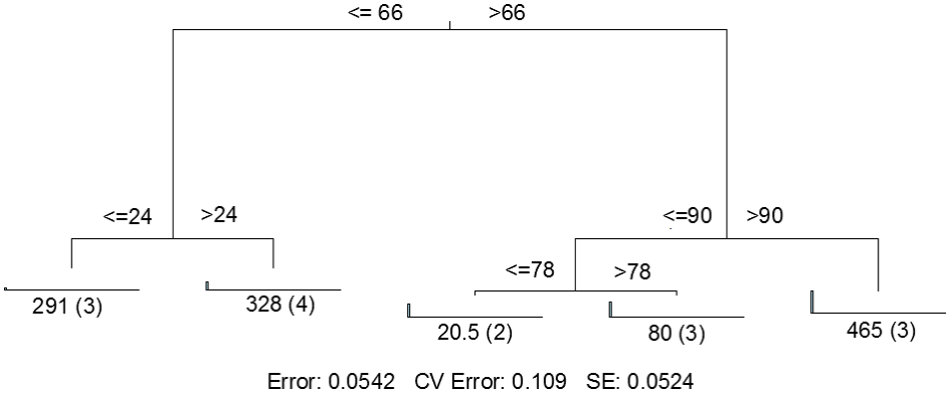
**Sum of squares multivariate regression tree representing data from Hurricane Sandy.** The response variable was viral community composition, and the explanatory variables were time and the environmental data set (Table 1). Cross-validation (100 times) was used to determine tree size. The five-leaf tree is formed by one major split and three smaller splits, all of which are based on time. Time values (in hours) are displayed on either side of each node. Numbers at each leaf represent the sum of squares of the response variable values about the node mean, and numbers in parentheses indicate the number of cases (samples) in each leaf. CV Error, cross-validated relative error, where 0 = perfect prediction and 1 = no predictive value.

MRT analysis revealed a clear relationship between time, rainfall, and viral community composition. RDA indicated that time was the single best explanatory factor in describing the viral community data (Table 3). Similarly, MRT indicated that time was the single best factor predictive of viral community composition (Figure 4). By cross-comparing the MRT with Figure 3, a clear relationship exists between the time intervals represented in the regression tree and storm stage (rainfall). The largest discontinuity in the viral community data occurred between 66 and 72 h, coinciding with peak rainfall, while the leaves of the tree represent pre-storm, rising intensity, falling intensity, and post-storm conditions (Figures 3, 4).

**Table 3:** **Results of redundancy analysis of viral community data in relation to environmental variables for Grim Dell retention pond during Hurricane Sandy (October, 2012) and Tropical Storm Andrea (June, 2013)**.

**Viral community data set**	**Variables selected by forward selection (*p* < 0.05)**	**Adjusted *R*^2^-value**	***F***	***p***	**Eigen value of axes**	**Variance explained by model (%)**
Hurricane sandy	Time	0.996	4556	0.001	1560	99
TS Andrea	Time	0.903	216	0.001	57.1	94

**Figure 5:**
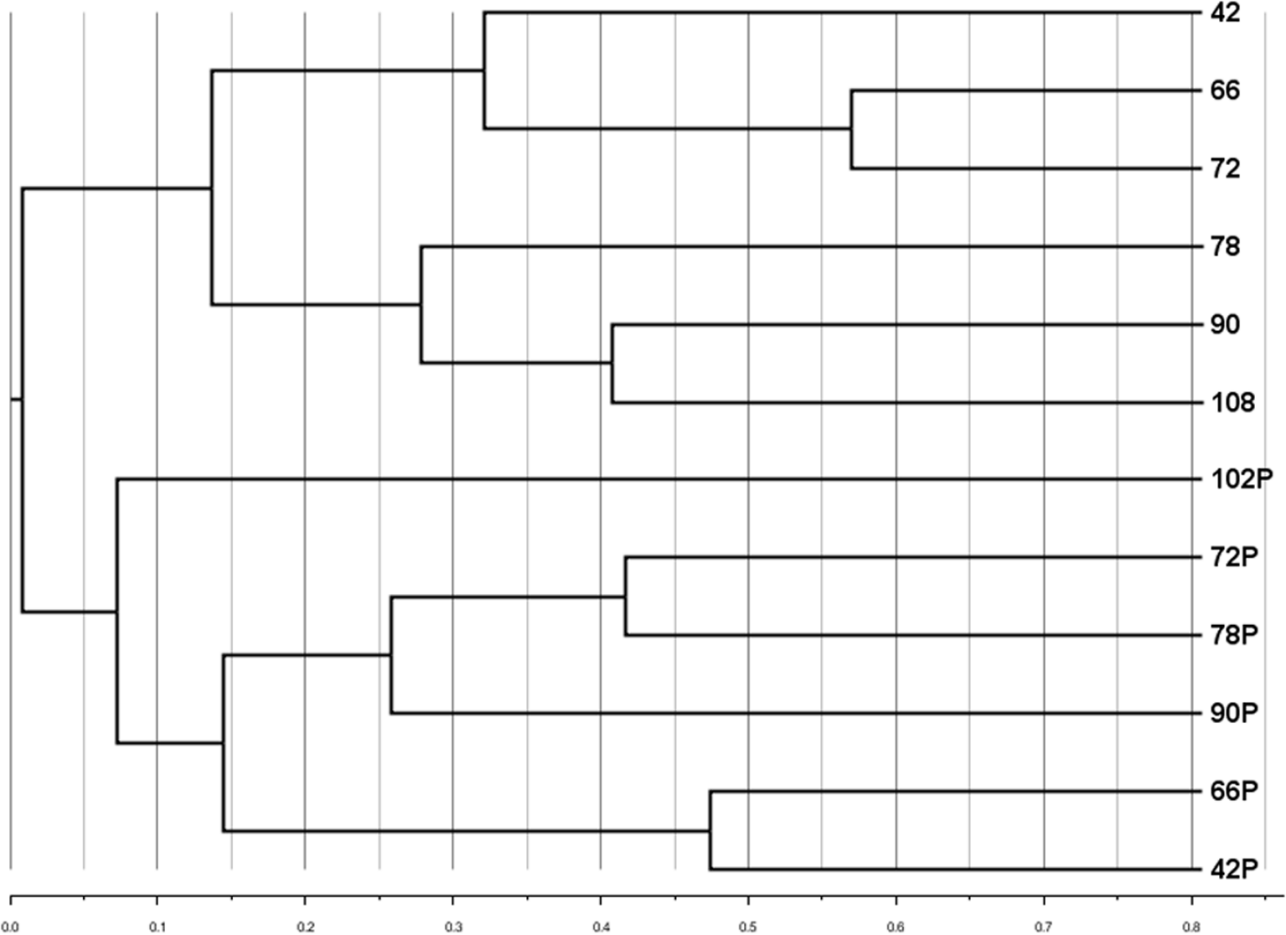
**Cluster dendrogram comparing composition of aquatic and particle-associated viral communities determined by RAPD-PCR banding patterns based on Dice similarity.** Samples are coded by the number of hours elapsed in the observation period; P designates particle-associated sample for each time point.

A subset of samples (those for which particle-associated abundances were determined, Figure 3A) was analyzed to compare the community composition of the particle-associated viruses. Comparisons of RAPD banding patterns across particle-associated VCs indicated that the composition of particle-associated viral communities in the Grim Dell changed over time (Figure 5). Furthermore, comparison of RAPD banding patterns between the particle-associated viruses and the planktonic viruses revealed that the two viral communities were completely distinct (Figure 5).

### Response of Grim Dell retention pond to tropical storm andrea

Surface water samples were collected twice per day from June 3–14, 2013. Although this sample interval captured changes in the pond due to Tropical Storm (TS) Andrea (June 6–11, 2013), several other rainfall events occurred during the observation period (Table 4). Rainfall varied from 0 mm over 48 h or more to >60 mm within 6-h during the peak of the tropical storm. Because of the unusually high frequency of rainfall events during June, samples were also collected July 16–19, 2013 during a dry period to compare changes in the pond in the absence of rainfall-runoff (Table 4). Over these two observation periods, pond pH varied from 5.08 to 7.19, with the highest reading at the beginning of the observation period and the lowest value following a week of dry weather. Surface water temperature was lower in the mornings and could be ≥5°C higher by the afternoon timepoint (approximately 6 h later) on the same day, likely due to the shallow depth of the pond. This daily variation was larger than the variation in the average temperature over the two observation periods. Conductivity was generally lower during rainfall events; however, decreases due to precipitation were not as dramatic as those observed during Hurricane Sandy in October, 2012 (Tables 1, 4). Dissolved oxygen increased during rainfall, most likely due to increased mixing during storms. TSS were generally higher during periods of rainfall and decreased with time following precipitation events, with notable exceptions (Table 4). For example, on June 11, 2013, 40 mm of rainfall had occurred since the time point 24 h prior, but TSS actually decreased over this period.

**Table 4:** **Environmental and community data for Grim Dell retention pond during Tropical Storm Andrea (June, 2013) and dry weather (July, 2013)**.

**Sample**	**pH**	**Temp (°C)**	**Conductivity (μS cm^−1^)**	**dO**_2_** (%)**	**TSS (mg L^−1^)**	**PO_4_ (μM)**	**NH_4_ (μM)**	**NO_2_ + NO_3_ (μM)**	**Partic P (μM)**	**Rainfall (mm)**	**V rich**
6-3_0830	7.19	21.4	479.2	23.5	125.1	0.29	13.58	*BDL*	0.40	0	12
6-3_1330	6.60	26.2	122.4	58.0	88.4	1.05	5.43	11.2	3.39	28.4	6
6-4_0930	6.76	21.8	127.3	45.0	55.0	1.57	5.62	10.5	2.26	5.6	4
6-4_1700	6.50	23.5	136.7	30.1	45.5	1.86	6.13	5.48	2.11	0	6
6-5_0930	6.65	20.9	143.9	26.6	44.0	1.86	7.12	6.65	1.93	0	7
6-5_1800	6.70	21.9	154.1	19.2	43.3	2.05	7.19	7.76	1.95	0	7
6-6_0940	6.74	20.9	168.1	33.1	29.1	1.52	6.39	4.76	1.73	0	13
6-6_1500	6.40	22.7	173.9	33.7	30.4	1.52	5.73	4.32	1.90	0	14
6-7_0930	6.10	21.9	109.8	77.0	23.6	9.04	3.71	5.60	1.83	36.6	15
6-7_1500	6.24	20.9	87.9	83.2	144.4	3.86	5.03	19.0	3.58	34.5	6
6-8_1300	6.40	24.0	105.7	48.2	213.1	7.38	3.67	8.31	6.96	61.2	7
6-9_1500	6.27	27.1	96.7	31.4	86.5	4.90	1.80	6.20	2.77	0	6
6-10_0930	6.53	23.5	101.5	25.0	198.9	5.00	4.29	4.27	7.13	0	11
6-11_0920	6.04	22.9	124.1	23.0	70.8	4.62	1.91	5.98	2.82	40.4	6
6-11_1500	5.98	27.5	130.3	28.0	90.3	4.90	2.20	2.49	3.67	0	6
6-12_0930	6.10	21.8	132.0	17.8	92.7	3.71	1.84	2.88	3.29	0	6
6-12_1430	6.54	26.2	136.2	33.4	73.3	3.52	BDL	2.27	3.98	0	8
6-13_0930	6.47	22.4	142.1	28.6	166.0	2.81	3.89	1.77	6.38	0	8
6-13_1520	6.50	34.0	152.9	38.0	124.5	1.38	BDL	0.06	7.00	0	10
6-14_0920	6.82	18.2	177.1	28.0	86.2	2.62	2.42	0.06	3.98	11.4	7
6-14_1500	6.72	21.0	178.9	32.1	64.8	3.33	2.31	1.11	3.26	0	ND
7-16_1010	6.17	24.4	127.2	28.6	63.7	3.5	2.5	5.5	2.60	0	ND
7-16_1330	6.08	26.4	127.5	31.2	58.6	2.9	2.8	5.0	2.70	0	ND
7-17_1010	6.09	24.6	162.6	34.2	43.3	3.7	1.1	3.7	4.29	0	ND
7-17_1330	6.02	28.7	156.2	52.4	68.0	2.5	2.1	3.1	4.15	0	ND
7-18_1010	6.61	24.5	211	48.0	68.0	6.2	5.1	2.5	7.08	0	7
7-18_1330	5.33	26.8	198.6	48.0	41.4	2.0	0.7	2.2	3.43	0	7
7-19_1010	5.80	23.1	302.3	32.0	52.0	2.1	2.0	1.2	6.07	0	8
7-19_1330	5.08	24.9	294.2	ND	53.4	1.2	1.5	1.7	3.52	0	6

All samples were collected in 2013. Samples are coded by m-d_hhmm; Temp, temperature; dO_2_, dissolved oxygen; TSS, total suspended solids; Partic P, particulate phosphorus; V Rich, viral richness; BDL, below detection limit; ND, not determined.

**Figure 6:**
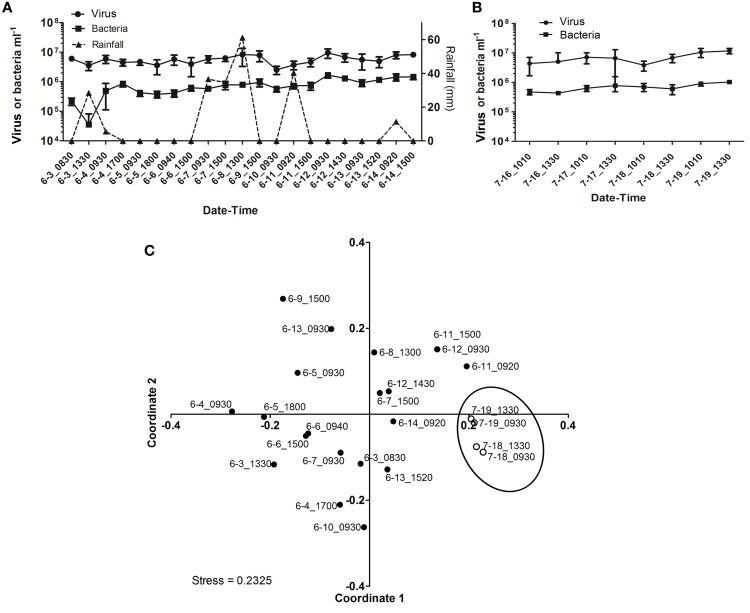
**Viral dynamics in Grim Dell retention pond during Tropical Storm Andrea and subsequent dry weather. (A)** Mean viral (closed circles) and bacterial (closed squares) abundances during Tropical Storm Andrea, error bars represent SD (*n* = 2); **(B)** mean viral (closed circles) and bacterial (closed squares) abundances during dry weather, error bars represent SD (*n* = 2); **(C)** non-metric multidimensional scaling (NMDS) plot of viral community composition as determined by RAPD-PCR banding patterns based on Dice similarity. Data points are coded by month-day-hhmm and correspond to samples listed in Table 4. Closed circles indicate samples collected during TS Andrea; open circles indicate samples collected during dry weather; circled area encompasses the dry weather samples.

As observed during Hurricane Sandy, viral and bacterial abundances did not change significantly due to precipitation and influx of stormwater runoff during TS Andrea (Figure 6A). Similar microbial abundances were observed under both wet (Figure 6A) and dry (Figure 6B) conditions, suggesting that rainfall-runoff does not significantly influence aquatic microbial abundance. Mann-Whitney *U*-tests were used to compare measurements between wet (June 3–14, 2013) and comparatively dry weather (July 16–19, 2013, Table 4). Viral and bacterial abundances and dissolved oxygen concentration were not significantly different in the pond during wet vs. dry weather. However, pH was significantly lower (*p* = 0.003) and surface water temperature and conductivity were significantly higher (*p* = 0.018, 0.010, respectively) during the dry weather observation period. Stepwise multiple regression analysis indicated that variation in viral abundance was best explained by the combined effects of conductivity and bacterial abundance, but the explanatory power of this model was much weaker than that for viral abundance during Hurricane Sandy (*R*^2^ = 0.57, *p* < 0.002; Table 5). Variation in bacterial abundance was best explained by combined effects of viral abundance, nitrate + nitrite, phosphate, and dissolved oxygen, with a more robust model than that for viral abundance (*R*^2^ = 0.70, *p* < 0.001; Table 5).

**Table 5:** **Stepwise multiple regression analysis of biological response variables in relation to environmental variables for Grim Dell retention pond during TS Andrea (wet + dry weather)**.

**Parameters**	**Adjusted *R*^2^**	***F***	**Coefficient**	***SE***	***p***
Viral abundance	0.579	5.531			0.002
BA			4.27	1.35	0.005
Cond			0.0006	2.825	0.047
Bacterial abundance	0.703	11.89			<0.001
VA			0.0021	0.0021	0.02
NO_2_ + NO_3_			0.0003	0.0008	<0.001
PO_4_			3.87	0.0001	0.02
dO_2_			0.0006	0.0002	0.03
Viral richness					ns
None					

Parameters defining the best performing models are shown.

BA, bacterial abundance; Cond, conductivity; VA, viral abundance; dO_2_, dissolved oxygen; SE, standard error; ns, not significant.

Viral community composition was compared based on RAPD-PCR banding patterns as described above. Band richness varied from 6 to 15 distinct band types (Table 4), but stepwise multiple regression analysis was unable to extract a useful model to explain variability in band richness in terms of environmental factors (Table 5). NMDS analysis of changes in RAPD banding patterns suggested that disturbance from storms induced changes in viral community composition (Figure 6C). Large shifts were associated with periods of rainfall (e.g., 6-3_0830 to 6-3_1330), and followed intense periods of rain (e.g., 6-8_1300 to 6-9_1500 to 6-10_0930; Figure 6C). Community composition dynamics for samples collected during dry conditions varied considerably from those collected during wet conditions. In the absence of precipitation, samples collected 4–24 h apart exhibited relatively little change in the viral community composition compared to any given pair of samples collected during wet conditions. A notable exception is the pair of samples 6-6_0940 and 6-6_1500 (Figure 6C), collected approximately 4 h apart during wet weather conditions. In this case, however, no rainfall was recorded for at least 48 h prior to sample collection and the small shift may represent a temporary stabilization in community composition.

**Figure 7:**
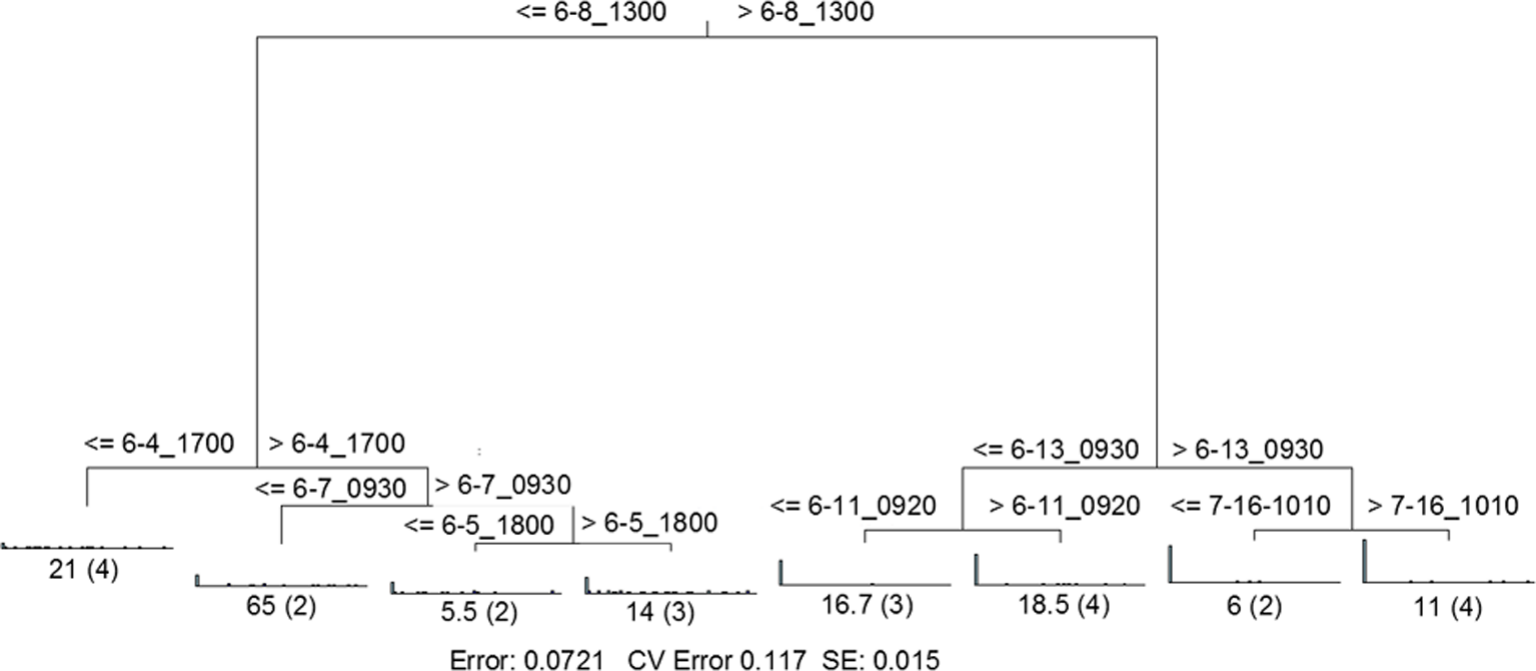
**Sum of squares multivariate regression tree representing data from Tropical Storm Andrea and dry weather.** The response variable was viral community composition, and the explanatory variables were time and the environmental data set (Table 3). Cross-validation (100 times) was used to determine tree size. The eight-leaf tree is formed by one major split and six smaller splits, all of which are based on time. Time values (in month-day_hhmm) are displayed on either side of each node. Numbers at each leaf represent the sum of squares of the response variable values about the node mean, and numbers in parentheses indicate the number of cases (samples) in each leaf. CV Error, cross-validated relative error, where 0 = perfect prediction and 1 = no predictive value.

RDA indicated that time was the single best explanatory factor in describing the viral community data (Table 3). As with the previous data set from Hurricane Sandy, MRT analysis indicated that time was the single best factor predictive of viral community composition during TS Andrea (Figure 7). Cross-comparison with Figure 6A reveals a clear relationship between the time intervals represented in the MRT and storm stage (rainfall). The largest discontinuity in the viral community data occurred at 6-8_1300, coinciding with peak rainfall during the observation period. With the exception of the discontinuity at 6-5_1800, all other leaves in the regression tree coincide with either changes in rainfall intensity or lack of rainfall. The discontinuity observed at 6-5_1800 may indicate further rearrangement of the viral community structure following the disturbance represented the pervious rainfall event.

## Discussion

This work represents the first known study to investigate the total viral load of stormwater runoff and measure the impacts of terrestrial runoff on freshwater aquatic viral communities. While this is an important first step in understanding how aquatic viral communities respond to infiltration of runoff, several important caveats must be taken into consideration. Microbial loads in runoff will differ based on catchment land use and land cover (Sharma et al., 2013), rainfall duration and intensity (Ran et al., 2013), antecedent catchment conditions and nutrient concentrations (McCarthy et al., 2013), as well as watershed area and the nature of the receiving water. In the present study, all field sampling was performed during major storm events and quantitative analysis of storm impacts on aquatic viral communities is limited to a single small catchment (stormwater retention pond). Thus, while it is highly likely that most rainfall-runoff events will transfer viruses and other microbes to aquatic habitats, the specific rates and impacts of such transfers will not be the same for all storm events or catchments.

Several previous studies in both marine and freshwater habitats have shown RAPD-PCR to be a robust method for documenting temporal changes in viral community composition (Winget and Wommack, 2008; Helton and Wommack, 2009; Winter and Weinbauer, 2010; Hardbower et al., 2012), hence its use in the present work. As in previous studies (Winget and Wommack, 2008; Helton and Wommack, 2009; Winter and Weinbauer, 2010; Hardbower et al., 2012), care was taken to ensure VCs were free of microbial contamination prior to RAPD-PCR amplification so that banding patterns originated from viral templates, and replicate PCRs yielded reproducible banding patterns of ≥90% similarity (Figure S1). While RAPD-PCR cannot provide information on the total viral community and only captures a subset of viral richness and dynamics (Winter and Weinbauer, 2010), RAPD-PCR is more sensitive to community changes and enables viral community profiling with smaller water samples than the alternative profiling approach, pulsed-field gel electrophoresis (Hardbower et al., 2012).

### Pilot study: CCC plantation

One drawback to our investigation of the microbial load carried by stormwater runoff at CCC Plantation is that the data set only represents a limited sampling of runoff during a single storm event. Furthermore, we did not attempt to pair microbial abundance estimates with environmental data. In spite of these limitations, this pilot study did provide the first known estimates of total viral abundances carried by stormwater runoff. Our data indicated that stormwater runoff can carry substantial numbers of viruses, with concentrations equivalent to many other aquatic habitats (Wommack and Colwell, 2000; Weinbauer, 2004; Clasen et al., 2008). Therefore, runoff-based transport of terrestrial viruses to aquatic ecosystems may influence temporal changes in the viral community composition of inland waters.

### Importance of particle-associated viruses

An important aspect of these pilot investigations of microbial abundances in stormwater runoff is estimation of the particle-associated fraction. Virus transport is known to be enhanced by binding to soil particles or aggregates, which themselves can be transported by mass flow (Chattopadhyay and Puls, 2000; Jin, 2000; Jin and Flury, 2002). However, relatively few studies have examined particle-associated microbes in natural aquatic systems, particularly freshwaters. In organic marine aggregates, viral abundances have been reported anywhere from 3 × 10^6^ to 8.7 × 10^10^ ml^−1^, comprising between 0 and 40% of the total viral abundance in the water column (Weinbauer et al., 2009). In a freshwater riverine system, the viral abundances associated with suspended solid material was reported to be 2 × 10^5^–5.4 × 10^9^ ml^−1^ (Luef et al., 2007, 2009), comprising 0.4–35% of the total viral abundance. In the present study, the abundances of particle-associated microbes were well within the range of previously reported values. However, in samples collected from stormwater runoff and from the Grim Dell during storm events, particle-associated viruses made up a higher percentage of the total viral abundance (up to 87.4%) than has been previously reported. If particle-enhanced transport of microbes is favored over the transport of suspended microbes during rainfall-runoff events, this could explain the higher percentages of particle-associated viruses observed in our samples.

To the best of our knowledge, this study is the first to include comparison of viral community composition between the planktonic and particle-associated fractions in any aquatic ecosystem. It is important to acknowledge the limitations in our analysis here: since only single samples (*n* = 1) of particle-associated viruses were prepared from each sample, our analysis cannot account for potential within-sample variability. Thus, actual differences between samples may be smaller than they appear. Bearing in mind this limitation, cluster analysis of viral RAPD-PCR banding patterns suggested that within a given water sample, the taxonomic composition of the particle-associated viral assemblage was almost completely different from that of the planktonic viral assemblage (Figure 5). The taxa represented by particle-associated viruses were not a subset or expanded set of planktonic viruses, but a virtually non-overlapping set. This observation would argue against equilibrium partitioning of viruses between the aquatic and sorbed phases. Given that the samples in the present study were collected during a major disturbance (storm), it is possible that more even distributions between planktonic and particle-adsorbed viral taxa can exist under calmer conditions or in other aquatic systems.

Particle-associated viruses may be important because particle surfaces are known to be hotspots for phage-host interactions (Mari et al., 2007; Samo et al., 2008; Weinbauer et al., 2009) that can play out in any number of ways: particle-bound viruses may attack planktonic hosts, particle-bound host cells may be attacked by planktonic viruses, or particle-bound microbes infected by particle-bound viruses may release new virus types into the water column. Furthermore, depending upon the size distribution and sinking rate of introduced particles, some particle-associated viruses may serve as a reservoir for future viral infections. Thus, the ultimate impacts of particle-associated viruses entering aquatic systems through stormwater runoff have yet to be determined.

### Response of planktonic viruses to rainfall-runoff events

Total viral and bacterial abundances in the Grim Dell did not vary more than 3-fold during storm events (Figures 3A, 6A,B). Although the CCC Plantation samples represent data from a different watershed, the viral abundances observed in stormwater runoff there were similar to the average viral abundance of the Grim Dell pond. If microbial concentrations in stormwater runoff are roughly equivalent to the receiving waters, then influx of microbes carried by stormwater would not significantly change aquatic microbial abundances per unit volume. While conductivity and bacterial abundance best explained variation in viral abundance during both observation periods, no shared factors were identified that best explained variation in bacterial abundance across the two observation periods (Tables 2, 5). Differences in the extracted explanatory variables across storm events may be due to differences in the storms, themselves. For example, the data collection period for Hurricane Sandy bracketed a single, well-defined precipitation event, while the data collection period for TS Andrea encompassed several discrete precipitation events. These differences in frequency and intensity of rainfall would most likely result in biological and chemical changes in the Grim Dell of differing magnitudes and time-scales. While we suspect rainfall is the ultimate cause of variation in the environmental factors (e.g., conductivity, pH, nutrient concentrations), rainfall was not extracted as a direct explanatory variable in the model, most likely due to the large number of zero values in both time series.

Some previous studies of freshwater systems have identified strong linkages between viral and heterotrophic bacterial dynamics (Personnic et al., 2009; Cheng et al., 2010; Hardbower et al., 2012), while others have found stronger correlations between viral dynamics and photosynthetic autotrophs (Madan et al., 2005; Clasen et al., 2008; Tijdens et al., 2008). Unfortunately, measurements of chl-*a* or other indices of photosynthetic biomass were not incorporated as part of our experimental design. However, we were able to test for correlations between viral and bacterial abundances across two storm events and a short dry weather period. While no clear relationship was observed between viral and bacterial abundances during Hurricane Sandy, during Tropical Storm Andrea and the subsequent period of dry weather, viral and bacterial abundances were significantly correlated to each other (Pearson *r* = 0.597, *p* = 0.001). These results suggest that rainfall-runoff can alter the linkages between aquatic viral and bacterial dynamics, but the impacts of individual storm events on microbial dynamics are clearly variable. Such variability is reasonable, given the differences in intensity and duration of the storms monitored.

Previous studies of temporal change in freshwater viral communities have typically described annual cycles of compositional change by comparing monthly samples (Filippini and Middelboe, 2007; Lymer et al., 2008; Tijdens et al., 2008). However, one study examined diurnal changes in freshwater viral community composition over 48 h, as well as at monthly intervals over 1 year (Lymer et al., 2008). In that study, the magnitude of diurnal changes could be as large as those of monthly changes within the same lake, suggesting that monthly sampling can underestimate the temporal variability in aquatic viral community dynamics. Our approaches in the present study represent more frequent and sustained sampling efforts, with samples gathered every 4–18 h for up to 10 consecutive days. The viral community composition in the Grim Dell was relatively stable over time periods of up to 48 h in the absence of rainfall-runoff (prior to Hurricane Sandy, Figures 3B, 4; dry weather conditions, Figures 6C, 7).

In at least some marine systems, microbial community dynamics exhibit reoccurring seasonal patterns in composition (Fuhrman et al., 2006; Gilbert et al., 2012; Parsons et al., 2012). Based on metagenomic analysis of marine prokaryotic communities, the reoccurrence of specific community structures in these studies has been attributed to changes in rank abundances of species that persist year-round. In other words, the taxonomic richness does not change over time, but changes in the rank abundance of individuals (distribution across taxa) leads to apparent changes in community composition (at least, as detected by coarser-grained approaches such as molecular profiling techniques), as well as changes in community function. The microbial communities of inland freshwater lakes may not share this feature, since some lakes exhibit regular annual recurrences of specific microbial taxa (Yannarell et al., 2003; Lymer et al., 2008) while others do not (Lindström, 2000; Boucher et al., 2006; Hardbower et al., 2012). The reasons behind this lake-to-lake variability are currently unclear, and indeed, the factors leading to temporal community change within any given lake remain, for the most part, poorly understood.

Our hypothesis is that changes in viral community composition observed in the Grim Dell were caused by the influx of novel virus taxa carried in stormwater runoff. This hypothesis is strongly supported by MRT analysis of viral community data. For both of the field observation periods in this study, the largest discontinuities in viral community structure coincided with peak precipitation, and the smaller discontinuities largely coincided with changes in precipitation intensity, i.e., increase, decrease, or cessation of precipitation (Figures 4, 7). This hypothesis is also supported by reports from other research teams focusing on freshwater bacterial communities, rather than viruses (Lindström, 2000; Lindstrom and Bergstrom, 2005). Most recently, analysis of the small subunit ribosomal gene sequences found in watershed soils, headwater streams, and a downstream lake (Toolik Lake, AK, USA) suggested that a substantial portion of bacterial taxa found in surface freshwaters originated from terrestrial environments (Crump et al., 2012). Thus, surface runoff from watershed soils may seed downstream water bodies with new microbial taxa, including viruses.

While this is a reasonable hypothesis, storm events entail multiple simultaneous processes that may affect aquatic viral community composition, and it is difficult to isolate the impacts of runoff-borne terrestrial microbial inputs against this background. For example, runoff may have introduced chemical inducing agents into the Grim Dell, inducing bacterial lysogens to release potentially novel viral genotypes into the water column. In the present study, dramatic changes in viral community composition were observed within time intervals as short as 4 h. Well-characterized lyogenic bacteria such as *Escherichia coli* (λ) can be induced to release prophage in time intervals as small as 2 h, at least under optimal culture conditions (Little et al., 1999). However, it is unclear whether prophage induction would proceed as quickly under environmental conditions, or indeed, whether prophage release can explain all the observed variability in our data. The observance of novel viral genotypes in the water column could also have arisen through release from infected (non-lysogenic) terrestrial bacteria that were carried along in stormwater runoff. Such release may still be interpreted as the transfer of terrestrial viruses to aquatic ecosystems; rather than being transferred as extracellular particles, viruses are simply transported in a vehicle (the infected cell) and released soon after arrival.

Finally, changes in aquatic viral community composition may also be driven by resuspension of viral particles from sediments. A recent study of two lakes in upper New York state found that virus genotypes typically associated with the water column as well as genotypes typically associated with watershed soils could both be found in lake sediments (Hewson et al., 2012). Since sediments could serve as a source of novel virus genotypes and the shallow depth of the Grim Dell (generally <2 m) would enable sediment resuspension during storms, we cannot rule out the possibility that changes in planktonic virus community composition were due to resuspension of viruses from sediments. In this scenario, changes in aquatic viral community composition would not be due to influx of terrestrial taxa as we hypothesized, but changes are still driven by storm impacts. While we have yet to determine the relative contributions of specific mechanisms (e.g., terrestrial runoff vs. sediment resuspension), disturbance due to storm events appears to strongly influence change in viral community composition in inland freshwaters.

Given the relationship between storm activity and aquatic viral community change, it is worthwhile considering these findings within the broader context of climate change. Most models predict increases in storm frequency and intensity in the mid-Atlantic region of the United States, where the present studies were performed (Ning et al., 2012). If the predicted changes in storm behavior are realized, this will translate to increased soil erosion, runoff, and transfer of microbes from soils to waterways, and may fundamentally change the microbial composition of inland freshwaters with unknown impacts on ecosystem function. In this regard, additional studies are needed to better characterize the impacts of storms on freshwater microbial community composition and subsequent impacts on function. Evaluating the variable impacts of stormwater runoff across different watershed types, storm durations, and storm intensities, as well as determining the ultimate impacts of these viral transport events on aquatic bacterial communities, represent important challenges for future research.

### Conflict of interest statement

The authors declare that the research was conducted in the absence of any commercial or financial relationships that could be construed as a potential conflict of interest.

## Supplementary material

Figure S1 **Comparison of fingerprints from randomly amplified polymorphic DNA (RAPD) polymerase chain reaction (PCR)**. Left half shows gel images generated by RAPD-PCR of **(A)** replicate samples and **(B)** DNase-treated samples. Viral concentrates were treated with 1 U μl^−1^ RQ1 DNase (Promega) according to manufacturer's instructions; in control reactions, DNase was replaced with sterile water. Sample names refer to time points in Table 1. R, replicate; POS, positive control; NEG, template-free control; L, molecular weight markers, indicated in base pairs of DNA; DNase, samples treated with DNase; Ctrl, DNase-free controls. Right half shows cluster dendrograms (Dice method) of **(C)** replicate samples and **(D)** DNase-treated samples.
