# Supplementary figures and images for: Stormwater runoff drives viral community composition changes in inland freshwaters
Source: Front Microbiol. 2014 Mar 14;5:105. doi: 10.3389/fmicb.2014.00105 (PMC3954104; doi:10.3389/fmicb.2014.00105)

a.

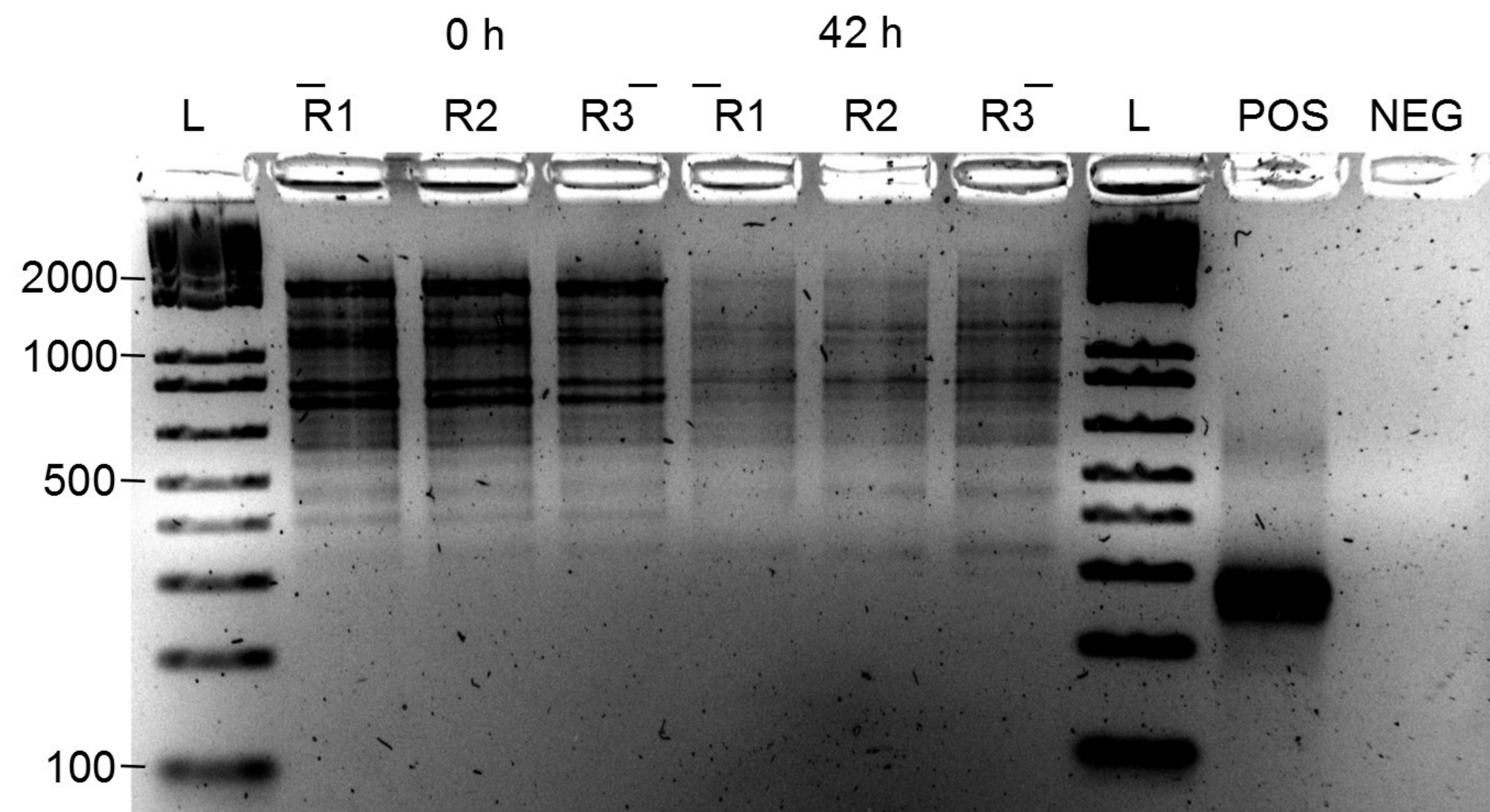

b.

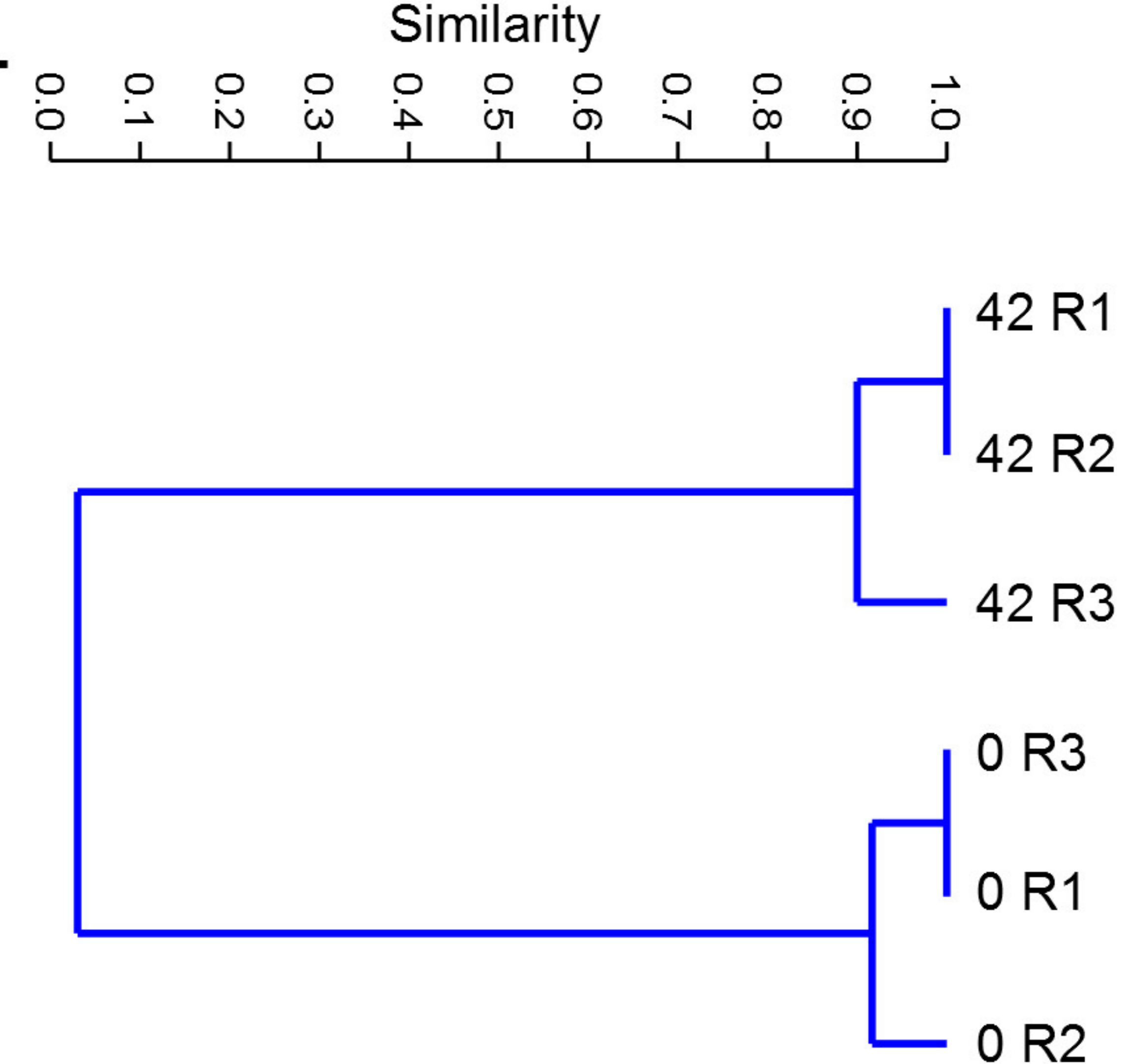

c.

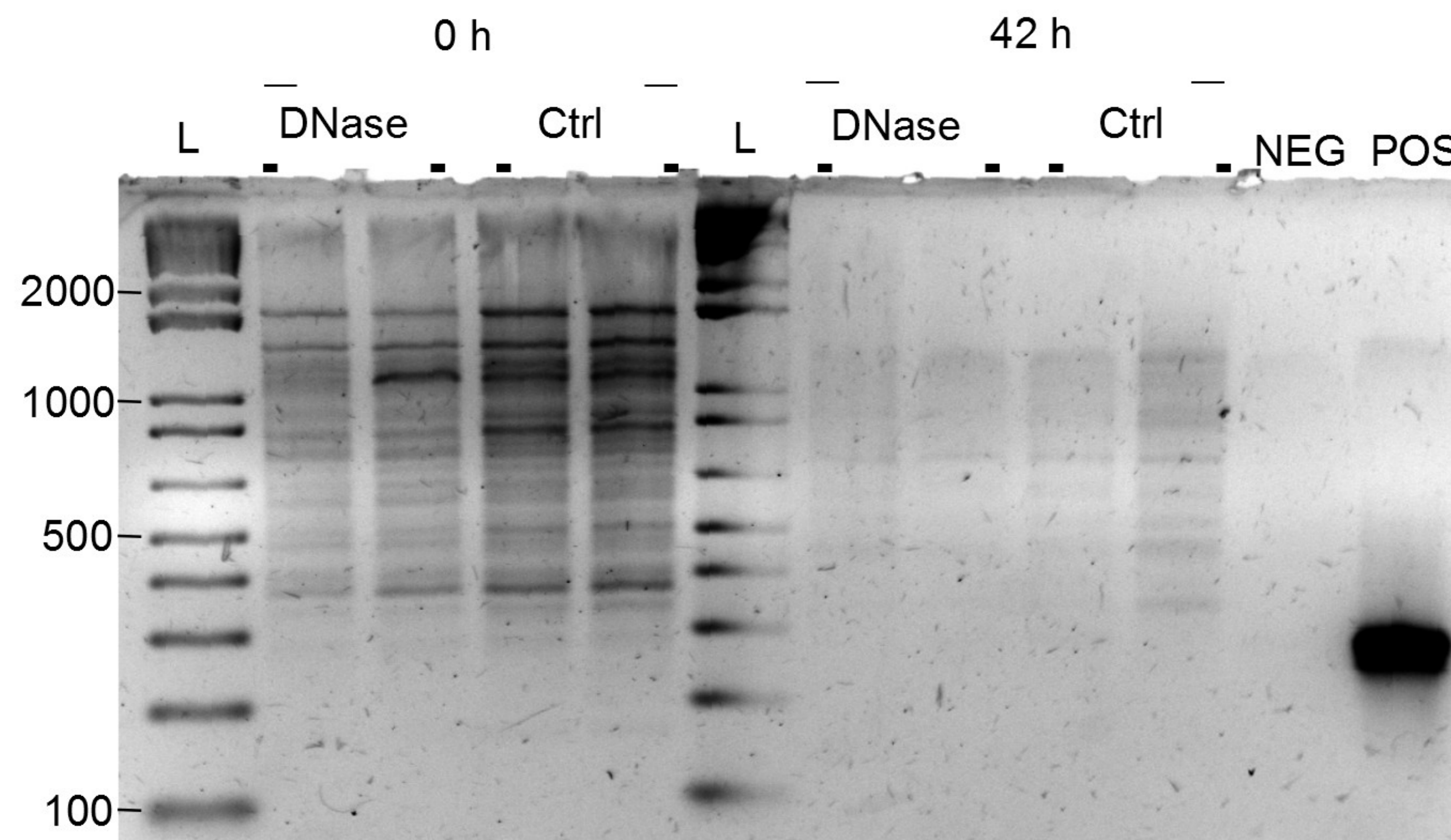

d.

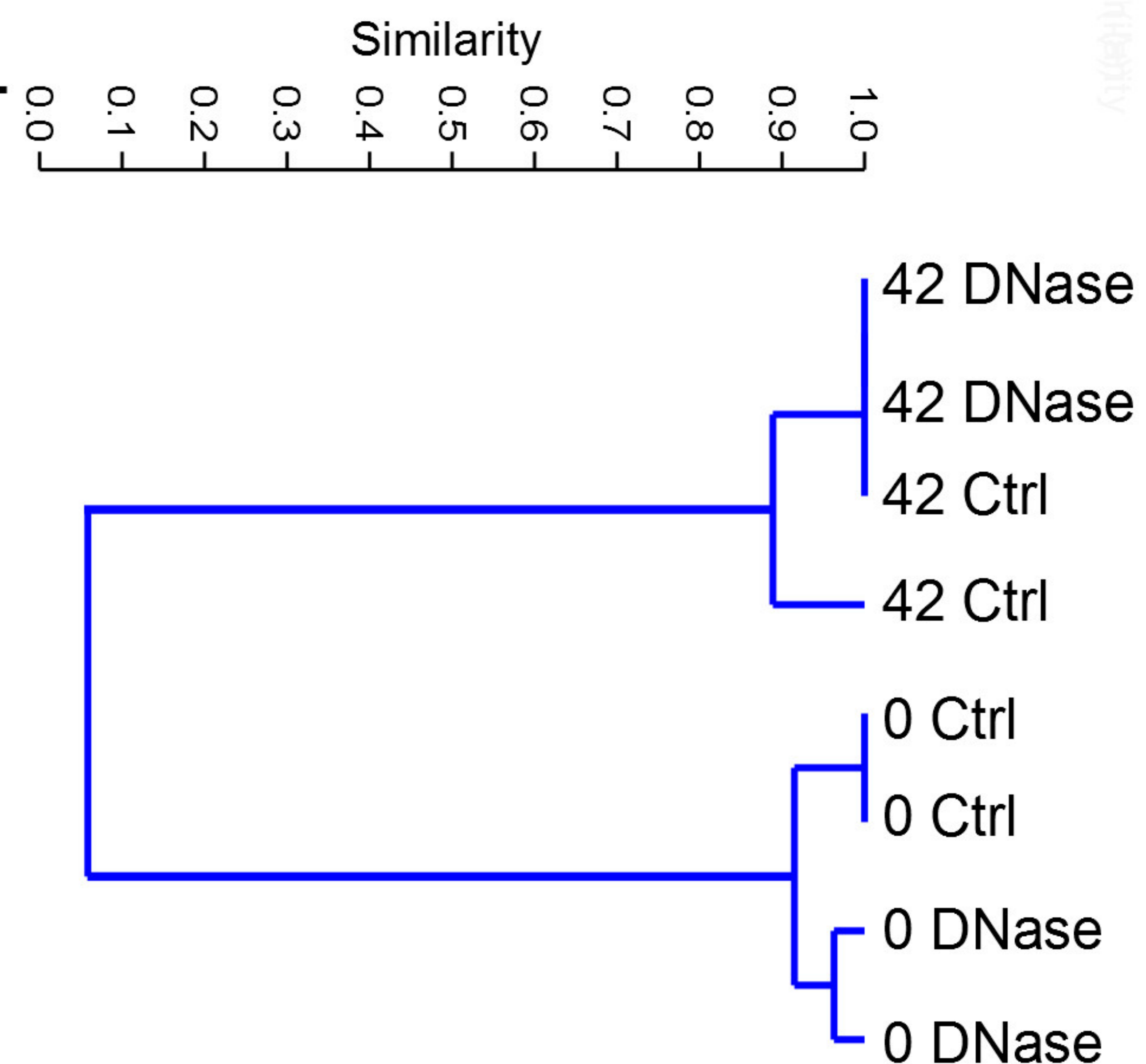

Supplement: Figure S1 — Comparison of fingerprints from randomly amplified polymorphic DNA (RAPD) polymerase chain reaction (PCR). Left half shows gel images generated by RAPD-PCR of (A) replicate samples and (B) DNase-treated samples. Viral concentrates were treated with 1 U μl−1 RQ1 DNase (Promega) according to manufacturer's instructions; in control reactions, DNase was replaced with sterile water. Sample names refer to time points in Table 1. R, replicate; POS, positive control; NEG, template-free control; L, molecular weight markers, indicated in base pairs of DNA; DNase, samples treated with DNase; Ctrl, DNase-free controls. Right half shows cluster dendrograms (Dice method) of (C) replicate samples and (D) DNase-treated samples. [file Presentation1.PDF]
